# Inducible and reversible *in vivo* protein degradation reveals T cell-intrinsic regulatory function of SHP-1

**DOI:** 10.3389/fimmu.2026.1961691

**Published:** 2026-10-21

**Authors:** Tabish H. Khan, Kodi S. Ravichandran, Ulrike M. Lorenz

**Affiliations:** 1 Department of Pathology & Immunology, Division of Laboratory and Genomic Medicine, Washington University in St. Louis, St. Louis, MO, United States; 2 Department of Microbiology, Immunology, and Cancer Biology, University of Virginia, Charlottesville, VA, United States; 3 Beirne B. Carter Center for Immunology Research, University of Virginia, Charlottesville, VA, United States; 4 Department of Pathology & Immunology, Division of Immunobiology, Washington University in St. Louis, St. Louis, MO, United States; 5 Bursky Center for Human Immunology and Immunotherapy, Washington University in St. Louis, St. Louis, MO, United States; 6 Unit for Cell Clearance in Health and Disease, VIB Center for Inflammation Research, Ghent, Belgium; 7 Department of Biomedical Molecular Biology, Ghent University, Ghent, Belgium

**Keywords:** auxin-induced degron, *in vivo* protein degradation, protein tyrosine phosphatase, SHP-1, T cell signaling

## Abstract

**Scientific problem:**

Studying the acute, cell-intrinsic functions of signaling proteins in T cells has long been challenging. Existing constitutive or inducible gene deletion cannot overcome developmental adaptation that obscures true phenotypes. Similarly, RNA interference suffers from incomplete knockdown and off-target effects, and pharmacological inhibitors often lack selectivity. Thus, approaches allowing rapid, specific, and reversible protein depletion in defined cell populations *in vivo* are needed.

**Methods:**

Here, we describe an Inducible and Reversible *in vivo* Protein Degradation (IRIP) system in T cells by combining auxin-induced degron technology with Cre-driver-mediated, lineage-specific expression of the plant E3 ligase adaptor OsTIR1. We first validated a minimal auxin-inducible degron tag (mAID-tag) inserted into the endogenous *Ptpn6* locus encoding the tyrosine phosphatase SHP-1 in murine and human T cell lines. We then generated *SHP-1^mAID^* knock-in mice and crossed them with mice carrying a floxed *OsTIR1* construct in the *Rosa26* locus, enabling Cre-mediated, T cell-specific, auxin-dependent SHP-1 degradation. We assessed *SHP*-*1^mAID^* protein degradation kinetics, specificity, reversibility, tissue restriction, and functional consequences.

**Results:**

The mAID-tagged SHP-1 protein was expressed at wild-type levels and retained full signaling function. Auxin treatment induced complete degradation of SHP-1mAID protein restricted to cells co-expressing OsTIR1, with no loss of untagged SHP-1. Degradation was fully reversible, with SHP-1 protein restored to baseline within 2–3 hours of auxin withdrawal *in vitro* and 60 hours *in vivo*. Degradation was confined to the T cell lineage by restricting OsTIR1 expression through a T cell-specific Cre driver. Such targeting could be extended to additional cell populations using a ubiquitous Cre driver. Acute *in vivo* depletion of SHP-1 caused T cell hyperproliferation upon restimulation, which was reversed upon SHP-1 re-expression.

**Conclusion:**

These findings demonstrate that SHP-1 restrains T cell proliferation in a manner that is direct, cell-intrinsic, acute, and independent of prior developmental conditioning. Our SHP-1 protein targeting approach also resolves a long-standing uncertainty in using the *motheaten* mouse line or the floxed *Ptpn6* locus. Our IRIP system represents a versatile and broadly adaptable platform for dissecting acute physiological roles of proteins whose constitutive loss produces complex and difficult-to-interpret phenotypes.

## Introduction

Over the last decade, significant progress has been made in studying T cell function and signaling through disruption of expression of a target protein and testing its function. However, all currently available methods have drawbacks. One of the most successful approaches has been to genetically target the deletion or mutation of specific genes, but once a gene is deleted or mutated, those modifications are permanent, and the cells can adapt to loss of a protein, thereby masking the phenotypes of interest. While transient introduction of siRNA minimizes the risk of cellular adaptation, transfection or transduction efficiency can be problematic and off-target effects remain a persistent concern (1, 2). The use of pharmacological inhibitors has proven useful for signaling studies given their often-immediate effects, but off-target activity and the drug entering multiple cell types are difficult to control (3, 4). Thus, there is a need for other approaches where the expression of a given protein can be ‘lost’ for a chosen period of time, and then reversibly the expression can be gained back. Ideally, such a system would allow rapid, reversible, and specific degradation of the target protein in the host cell.

One technology that fulfills these criteria is the auxin-induced degron (AID) system. Auxins are a class of plant hormones that bind to the plant protein Transport Inhibitor Response 1 (TIR1). The auxin-TIR complex, in turn, binds proteins that contain the AID targeting sequence or its minimal 68 amino acid sequence, referred to as mAID. This triggers rapid polyubiquitylation and proteasomal degradation of the mAID-containing target protein (5, 6). This AID system has been successfully adapted for use in mammalian cells (6–8). Although the AID-targeting region/mAID sequence is not found in mammalian cells, the degradation feature is transferable: when the mAID sequence is fused to a protein of interest, it can direct its degradation in the presence of auxin and TIR. The TIR1 protein (usually rice-derived version called OsTIR1) forms an SCF (Skp1-Cullin-F-Box) E3 ubiquitin ligase complex with endogenous cellular components, and in this configuration the complex does not target any endogenous proteins. However, as in plants, auxin addition causes proteins bearing the AID targeting sequence to be recruited into the TIR1-SCF complex, resulting in their degradation. While the original full-length AID region has been reported to cause protein degradation independently of OsTIR1 or auxin, shortening the AID domain to a minimal 68 amino acid sequence rendered degradation faithfully controllable (9).

SHP-1 (encoded by the *PTPN6* gene) is a non-receptor protein tyrosine phosphatase predominantly expressed in hematopoietic cells (10, 11). It functions as a critical negative regulator of intracellular signaling by dephosphorylating key tyrosine residues on receptors and signaling intermediates (12–15). Under basal physiological conditions, SHP-1 maintains a relatively stable expression profile with a half-life of 12–24 hours (16), and its catalytic activity is governed by Src homology 2 (SH2) domain engagement and post-translational modifications (11, 17, 18). However, specific post-translational modifications (19) or pathophysiological states can trigger its ubiquitination and subsequent proteasomal degradation (20). Through its phosphatase activity, SHP-1 modulates immune cell activation, proliferation, and tolerance (21–25). Despite being a well-described negative regulator of T cell receptor signaling with established roles in T cell development and function (26–29), there remains controversy as to whether the *in vivo* phenotypes observed in SHP-1-deficient mice reflect direct effects on T cell receptor signaling or are instead the result of *in vivo* adaptation to chronic SHP-1 deficiency (25, 30). Constitutive deletion of SHP-1 in T cells does not allow these possibilities to be distinguished, as the protein loss occurs throughout development and the cells could rewire their signaling networks long before any functional readout is assessed.

To address this, we developed a system we term *Inducible and Reversible in vivo Protein Degradation*, or IRIP, by combining the auxin-induced degron system for protein degradation with lineage specificity and temporal control achieved through existing Cre driver strains. We applied IRIP to SHP-1 to evaluate the system in the context of a well-characterized signaling pathway while simultaneously generating a useful tool for future studies. Using this approach, we directly assess the regulatory role of SHP-1 in T cells through short-term, temporally controlled depletion and find that SHP-1 directly limits the extent of T cell proliferation in a cell-intrinsic and reversible manner, resolving the outstanding question of whether this phenotype is a consequence of acute signaling regulation or of prior developmental conditioning.

## Materials and methods

### Mice

*SHP-1^mAID^* mice were generated in the C57BL/6 background using CRISPR-Cas9–mediated knock-in technology (**Supplementary Figures 1A–C**). Knock-in was confirmed by sequencing, PCR genotyping (**Supplementary Figure 1D**) and protein expression. Heterozygous *SHP-1^mAID^* mice were crossed to homozygosity and used in all studies, unless otherwise indicated. Transgenic mice carrying a Cre-inducible OsTIR1 transgene (31) were generated by targeted insertion of the transgene into the Rosa26 locus using homologous recombination-mediated knock-in strategies. Correctly targeted founder animals were identified by PCR genotyping (**Supplementary Figure 1D**) and subsequently bred to establish stable transgenic lines on the C57BL/6 background. OsTIR1 expression was driven in the T cell lineage through crossing with mice expressing Cre under the control of the distal Lck promoter (dLck-Cre) (32) or ubiquitously through crossing with the deleter EIIA-Cre strain (33) that expresses Cre under the control of the adenovirus EIIa promoter driving expression of Cre recombinase to the early mouse embryo, both of which were purchased from the Jackson Laboratory (Bar Harbor, ME). 8-week-old mice were used throughout these studies, unless specified otherwise. All experimental mice are age and sex matched and housed and bred in the specific pathogen-free facility at the Washington University in St. Louis. Experiments were approved by the Animal Care and Use Committee of the Washington University in St. Louis.

### Generation of SHP-1^mAID^ knock-in T cell lines

Parental murine BYDP cells (34) and human Jurkat cells (35) were cultured as previously described (36). To knock the 68 aa mAID domain (7) into the endogenous SHP-1 C-terminus, the following strategy was developed. A self-linearizing mAID knock-in construct encoding the 68 aa mAID encoding sequence fused to genes encoding Puromycin or Blasticidin selection markers as well as mCherry or EGFP fluorescent proteins was cloned into pcDNA 3.1 (Addgene # V790-20) backbone. P2A and T2A cleavage sites were incorporated between proteins. The insert was bordered by 2 kbp 5’ and 3’ SHP-1-derived flanking sequences. To increase knock-in efficiency, a vector-targeting self-linearizing sgRNA was cloned upstream (**Supplementary Figure 2A**). The SHP-1 sgRNA (**Supplementary Figures 2A, C**) was cloned into the S. pyogenes Cas9 plasmid (Addgene #42230). Myc-tagged OsTIR1 gene (Addgene #47328) was cloned into a derivative of the pBabe plasmid (Addgene #1764), where the Puromycin resistance gene had been replaced with Zeocin resistance gene (pGIPZ) (**Supplementary Figure 2A**). To generate T cell lines that constitutively express OsTIR1, BYDP and Jurkat T cell lines (10^7^ each) were transduced with OsTIR1-pBabe followed by selection with Zeocin at 200-400 μg/ml for 5 days. OsTIR1-positive as well as parental BYDP and Jurkat T cell lines were then electroporated with the two different mAID encoding constructs (4µg each) and the sgRNA *S. pyogenes* Cas9 plasmid (2 µg) followed by dual selection with Puromycin (0.5-10 µg/ml Jurkat and BYDP respectively, CAS Number: 58-58-2, InvivoGen) and Blasticidin (15-20 µg/ml Jurkat and BYDP respectively, CAS Number: 3513-03-9, InvivoGen).

### T cell stimulation

Parental and newly generated derivative cell lines of BYDP and Jurkat T cells were stimulated as previously described (25, 37). Briefly, cells were stimulated through a TCR/CD3ϵ complex and CD4 receptors by using 1.0 μg/ml anti-CD3ϵ (145-2c11 [Cedarlane] for BYDP-derived cell lines or OKT3 [BioLegend] for Jurkat-derived cell lines) in combination with 0.5 μg/ml anti-CD4 (Leu3a, BD Biosciences) followed by cross-linking with 10 μg/ml goat anti-mouse IgG (Southern Biotechnology Associates). After incubation at 37 °C for the indicated times, cells were washed twice in cold phosphate-buffered saline (PBS) containing 1 mM Na_3_VO_4_ and lysed in 250 µl RIPA buffer (NaCl 150 mM, Tris 50 mM, Nonidet P-40 1%, sodium deoxycholate 0.5%, SDS 0.1%), supplemented with protease and phosphatase inhibitor at 4 °C (Cat. No. 78440; Thermo Fisher), followed by analyses via Western blot.

### Administration of auxin (IAA)

#### Cell culture studies

For the treatment of BYDP and Jurkat cell lines, auxin (Indole-3-Acetic Acid) was obtained from RPI (CAS Number: 87-51-4), dissolved in dimethyl sulfoxide (DMSO, Sigma-Aldrich) to create a 0.05 M stock solution, which was stored at −20 °C and used as indicated.

#### Primary cell and *in vivo* studies

Because of the toxicity of DMSO to primary cells, all primary cell and *in vivo* applications were performed with PBS-soluble auxin, obtained from Cayman Chemical (CAS No. 6505-45-9) at the indicated concentrations. For a comparison of the biological activities, refer to **Supplementary Figure 3**. For *in vivo* applications, auxin was freshly dissolved in 200 μl of PBS at indicated concentrations and administered intraperitoneally.

### Isolation of primary T cell and non-T cell populations

Lymph nodes and spleens were harvested from 8-week-old mice. Tissues were mechanically dissociated and filtered through 70 µm cell strainers (Cat. No. 130-098-462; Miltenyi Biotec). For the splenic samples, red blood cells (RBCs) were lysed using an RBC lysis buffer (Cat. No. 00-4333-57; Invitrogen). CD4^+^ and Pan-T cells were isolated using magnetic bead-based negative selection kits for CD4^+^ (Cat. No. 130-104-454; Miltenyi Biotec) or Pan-T cells (Cat. No. 130-095-130; Miltenyi Biotec) per the manufacturer’s instructions. Splenocytes were labeled with the provided antibody cocktail, and T cells were obtained by negative selection using LS columns. Non-T-cell populations were isolated via positive selection from the cell fraction retained on the column during negative selection of Pan-T cells.

### Flow cytometry analysis

Flow cytometry data for BYDP and Jurkat T cells co-expressing mCherry and GFP tracker, were acquired on a Attune NxT flow cytometer and analyzed using FlowJo v10 software. CD4+T cells isolated from spleens of *SHP-1^mAID^* mice were stained with surface staining marker CD4 (Cat. No. 17-0042-83; Invitrogen) in FACS buffer (1% BSA and 0.5mM EDTA). Fixable live/dead staining (Cat. No. L35959; Thermo Fisher) was performed after surface staining in PBS.

### Western blot

Isolated cells with or without stimulation were washed with PBS and lysed with NP-40 buffer (NaCl 150 mM, Tris 50 mM, Nonidet P-40 1%, sodium pyrophosphate 4mM) or RIPA buffer (NaCl 150 mM, Tris 50 mM, Nonidet P-40 1%, sodium deoxycholate 0.5%, SDS 0.1%) supplemented with a protease and phosphatase inhibitor cocktail (Cat. No. 78440; Thermo Fisher) as previously described (22). Lysed samples were spun down at 10,000 g to remove debris and boiled at 95 °C for 10 min, with Tris-Glycine buffer (SDS 2%, Bromophenol Blue 0.01%) containing 0.1 M DTT. Normalized amounts of protein were loaded onto a 10% acrylamide gel and run in Tris-glycine running buffer (25 mM Tris, 192 mM glycine, (pH 8.3)). Gels were electro-blotted onto PVDF membrane in CAPS transfer buffer (10 mM 3-(cyclohexylamino)-1-propanesulfonic acid, 10% methanol (pH 11.0)). Membranes were blocked 45 min. at room temperature in 5% milk powder (Marvel) in TBS-T (TBS, 0.1% Tween-20). Blots were probed with anti-phosphotyrosine (clone 4G10 1mg/ml; Millipore), pLAT (Cat. No. 3581S; Cell Signaling Technology), monoclonal anti–SHP-1 (clone 1SH01 1:300; Thermo Fisher Scientific) and/or anti-Myc tag (clone 9E10 1:300; Thermo Fisher Scientific, Waltham, MA) and reprobed for β-actin as a loading control (anti–β-actin-HRP, clone AC-15 1:30.000; Abcam). Blots were imaged using the ChemiDoc Touch gel imaging system (Bio-Rad Laboratories, Hercules, CA).

### T- cell proliferation assay

CD4+ cells were isolated from the spleens of *SHP-1^mAID^: OsTIR+:dLckCre+* and *SHP-1^mAID^: OsTIR****-****:dLckCre****-*** control mice that had been injected with 600mg/kg of auxin as specified. *SHP-1^mAID^: OsTIR+:dLckCre+* injected with PBS were used as control. 2.5x10^5^ CD4+ T cells were labeled with 5μL CellTrace Violet (Cat. No. C34557; Invitrogen), washed and stimulated *in vitro* with plate-bound αCD3 (0.25–1.0 µg/mL) and αCD28 (1.0 µg/mL) for 72 hours in 200μL 1640-RPMI complete media (RPMI, Gibco; supplemented with 10% heat inactivated FBS (Avantor Seradigm), 2mM L-glutamine, 10mM HEPES, 1mM MEM sodium pyruvate, 100μM non-essential amino acids, 50μM 2-mercaptoethanol, and concentration penicillin streptomycin) in 96-well flat bottom plates. After 3 days, cultures were harvested and prepared for flow cytometric analyses. Proliferation was calculated based on CellTrace Violet staining using the proliferation analysis tool in FlowJo v10, as previously described (22, 25).

### Statistical analysis

Statistical analysis was performed using one-way ANOVA (GraphPad Prism 10) to compare differences among the experimental groups. A p value of <0.05 was considered statistically significant, and all data are presented as mean ± SEM. Asterisks represent *p<0.05, **p<0.01, ***p<0.001, ****p<0.0001.

## Results

### Generation and characterization of an *in vitro* auxin-inducible and reversible protein degradation system in murine and human T cell lines

To develop the auxin-induced degron (AID) system, we chose to target SHP-1, a well-described critical negative regulator of T cell receptor signaling (15, 21), as this would allow us to evaluate the system in the context of well-characterized signaling pathways while simultaneously generating a useful tool for future studies. The overall strategy is illustrated in **Figure 1A**. We initially established the parameters of IRIP using mammalian T cell lines to assess the feasibility of the approach and the functional integrity of the tagged protein before proceeding to the generation of mouse strains. We chose two T cell lines for the *in vitro* development of the system: the murine BYDP T cell line (38), which we have studied extensively to explore the function and biochemistry of SHP-1 (39) and which would facilitate the transition to *in vivo* mouse studies, and the human Jurkat T cell line (35), which is widely used for studies of T cell signaling including those involving SHP-1 (40).

**Figure 1:**
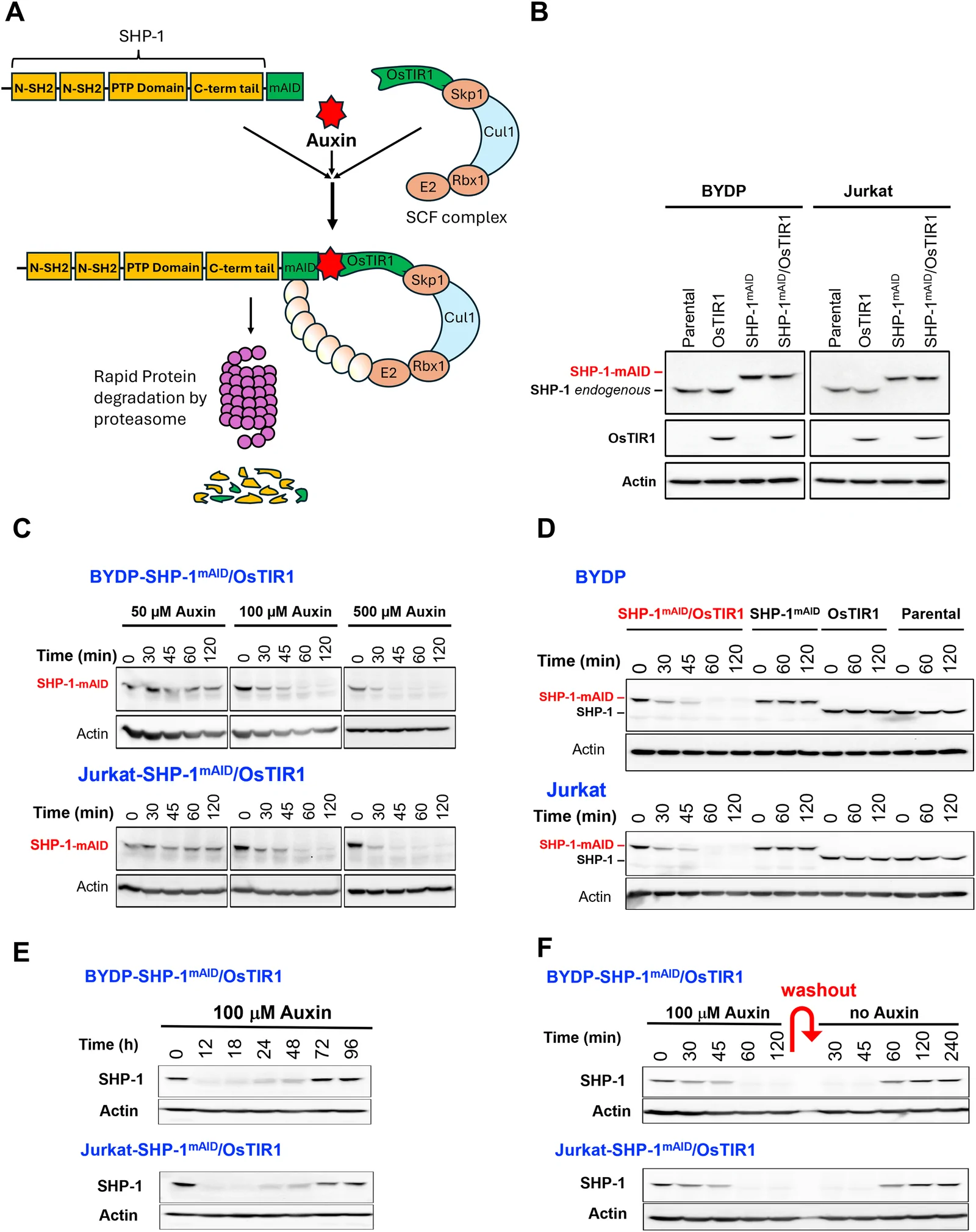
Generation and validation of an *in vitro* auxin-inducible degron system for SHP-1 in murine and human T cell lines. **(A)** Schematic of the AID system. The target protein SHP-1 is C-terminally fused to a mini-AID (mAID) degron. In the presence of auxin, the plant-derived F-box protein OsTIR1 binds the mAID-tagged SHP-1 and recruits it to the SCF E3 ubiquitin ligase complex for ubiquitination and subsequent degradation by the 26S proteasome. Orange indicates endogenous tagged protein; green indicates plant-derived proteins/domains; and light orange and light blue indicate endogenous components of SCF E3 ubiquitin ligase complex. **(B)** Immunoblot analysis of equal aliquots of whole-cell lysates from engineered BYDP and Jurkat cells validates the expression of the SHP-1^mAID^ fusion protein and OsTIR1. Blots were probed with antibodies against SHP-1 (detecting endogenous and tagged forms), myc-tag (for OsTIR1), and β-actin as a loading control. Data are representative of at least three independent experiments. **(C)** Kinetics and dose response of auxin-induced SHP-1 degradation. Engineered BYDP and Jurkat cells expressing SHP-1^mAID^ and OsTIR1 were treated with the indicated doses of auxin for 0–2 hours. SHP-1 protein levels were assessed by immunoblotting at the indicated time points. β-actin serves as a loading control. **(D)** Auxin-induced degradation is dependent on OsTIR1 expression and the mAID tag. Degradation of SHP-1 is only observed in cell lines expressing both SHP-1^mAID^ and OsTIR1. **(E)** Long-term kinetics of SHP-1 degradation in cells cultured with a single dose of auxin without replenishment. **(F)** Reversibility of the AID system. After auxin treatment, cells were washed and cultured in auxin-free medium. Immunoblot analysis for SHP-1 demonstrates that protein expression is restored following auxin washout. All immunoblots are representative of three independent experiments.

Endogenous SHP-1 was tagged at the carboxyl terminus with mAID (31) using CRISPR/Cas9 (41), as we have previously shown that C-terminal fusions preserve SHP-1 function (38). To ensure that both alleles were tagged, two different selection vectors were used, enabling double selection with puromycin and blasticidin (**Supplementary Figure 2A**). Genes encoding mCherry and GFP were additionally introduced to allow straightforward identification of engineered cells by flow cytometry (**Supplementary Figures 2A, B**). To avoid propagation of additional mutations during single cell cloning, bulk populations were used for all subsequent studies. OsTIR1, derived from rice *Oryza sativa*, was then introduced into the genetically modified BYDP and Jurkat cell lines to mediate auxin-induced degradation of SHP-1^mAID^. A carboxyl-terminal myc-tag was added to OsTIR1 for easy protein detection, and successful *SHP-1^mAID^* knock-in and OsTIR1 expression in the engineered cell lines were confirmed by immunoblotting (**Figure 1B**).

Having successfully generated the knock-in T cell lines, we next determined the optimal auxin dosage and characterized the kinetics of protein degradation. Auxin treatment at concentrations of 50 µM, 100 µM, and 500 µM resulted in dose-dependent loss of SHP-1^mAID^ protein in both BYDP and Jurkat cells. While 50 µM auxin did not induce detectable SHP-1^mAID^ degradation, treatment at 100 µM and above resulted in measurable degradation within 30 minutes, with complete loss of protein observed by 60 minutes (**Figure 1C**), confirming that auxin-induced degradation operates in a dose- and time-dependent manner.

To assess the specificity of the system, we compared SHP-1 protein levels across a panel of control cell lines, those expressing only SHP-1^mAID^, only OsTIR1, or neither, following auxin treatment. SHP-1 degradation occurred exclusively in cells co-expressing both SHP-1^mAID^ and OsTIR1 (**Figure 1D**), with no loss of endogenous SHP-1 detected in any control line at any dose or time point examined.

We next assessed the long-term kinetics of auxin-induced degradation and the reversibility of the system. In BYDP and Jurkat T cells cultured following a one-time treatment of 100 µM auxin, SHP-1^mAID^ remained below the level of detection for up to 12 to 18 hours. Partial re-expression was observed between 24 and 48 hours, and full re-expression was achieved by 72 to 96 hours in both cell types without auxin replenishment (**Figure 1E**), indicating that degradation is sustained until auxin is metabolized or depleted from the culture medium. To specifically characterize re-expression kinetics following auxin withdrawal, cells were treated with 100 µM auxin for 2 hours, washed twice, and reseeded in fresh medium. SHP-1^mAID^ protein was detectable within 1 hour of washout and reached full baseline expression levels within 2 to 3 hours (**Figure 1F**), demonstrating the rapid and controllable nature of protein modulation afforded by this system.

### C-terminal mAID fusion preserves SHP-1 signaling function

To determine whether the C-terminal fusion of mAID to SHP-1 might affect its function in T cells, we assessed the TCR-proximal signaling downstream of TCR/CD4 stimulation. We have previously shown that BYDP cells rendered deficient in SHP-1 activity display increased total tyrosyl phosphorylation as well as enhanced phosphorylation of LAT, ZAP-70, and Lck upon TCR/CD4 stimulation (34). We therefore examined total tyrosyl phosphorylation in murine BYDP and human Jurkat T cell lines expressing wild-type SHP-1, SHP-1^mAID^ alone, or SHP-1^mAID^ together with OsTIR1. Parental BYDP and Jurkat cells pre-treated for 8 hours with or without auxin (**Figures 2A, D**), as well as control lines expressing OsTIR1 or SHP-1^mAID^ alone (**Figures 2B, C, E**, left panels), were stimulated through TCR/CD4 for the indicated length of time as previously described (34), and cell lysates were analyzed for induced tyrosine phosphorylation. The pattern and extent of phosphorylation were comparable across all control conditions, indicating that neither the mAID tag nor OsTIR1 expression alone perturbs TCR/CD4 signaling.

**Figure 2:**
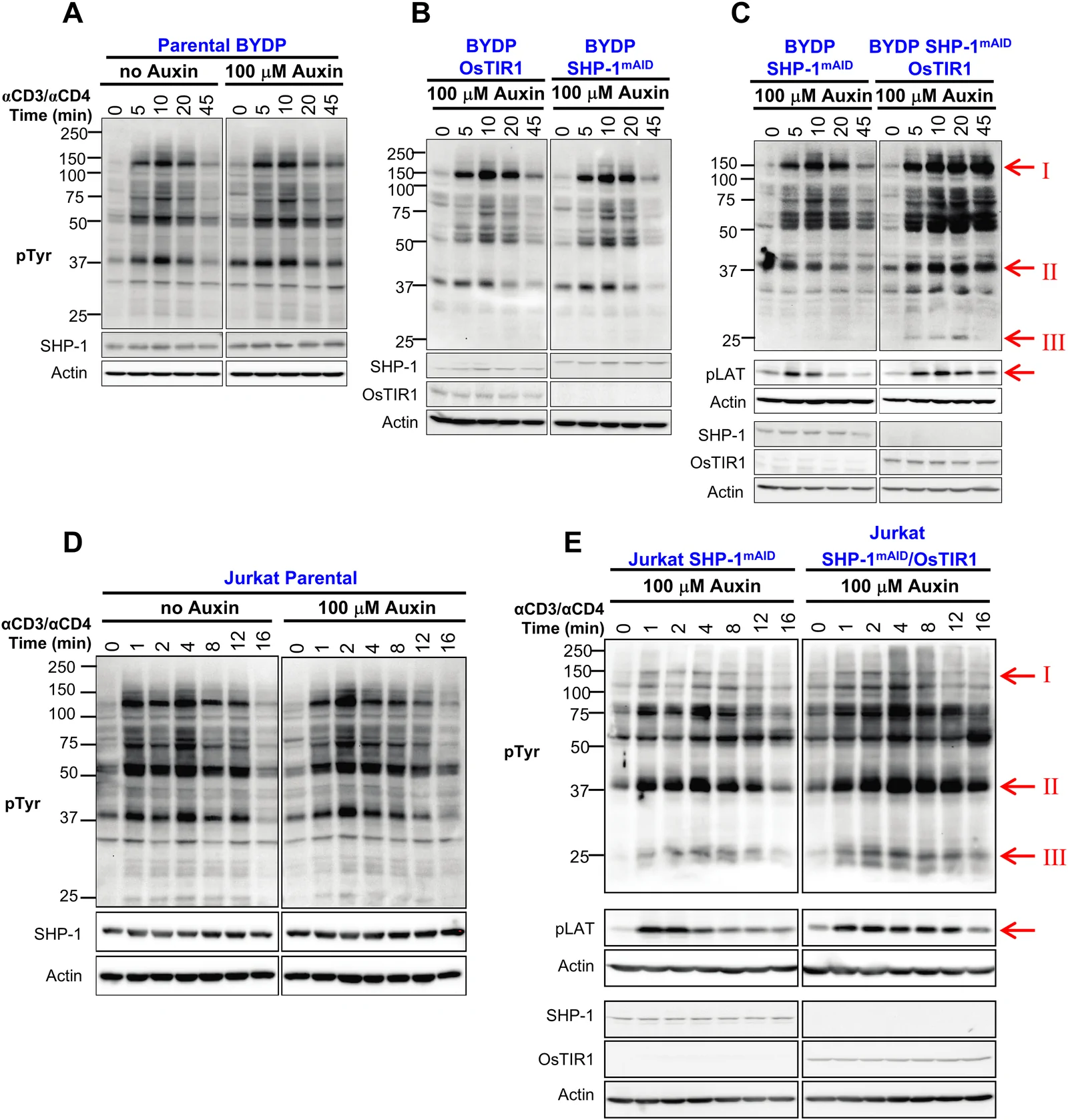
C-terminal fusion of mAID to SHP-1 does not impair its function in TCR signaling. Immunoblot analysis of phospho-tyrosine (pTyr) levels in key signaling proteins following αCD3/αCD4 stimulation in murine (BYDP) and human (Jurkat) T cell lines. Cells expressing SHP-1, SHP-1^mAID^, or both SHP-1^mAID^ and OsTIR1 were pre-treated with auxin for 8 hours to induce SHP-1 degradation. **(A)** Parental BYDP cells. **(B)** BYDP cells with *SHP-1^mAID^* knock-in. **(C)** BYDP cells with *SHP-1^mAID^* and *OsTIR1*. **(D)** Parental Jurkat cells. **(E)** Jurkat cells with *SHP-1^mAID^* and *OsTIR1*. Red arrows indicate increased phosphorylation of key proteins (I-III) upon SHP-1 degradation, specifically in cells expressing both SHP-1^mAID^ and OsTIR1, confirming that the mAID tag does not interfere with SHP-1’s phosphatase function. β-actin and total SHP-1 and/or OsTIR1 protein levels serve as loading controls. Data are representative of three independent experiments.

In contrast, in BYDP and Jurkat cells co-expressing SHP-1^mAID^ and OsTIR1 stimulated following auxin pre-treatment, consistent with the loss of SHP-1 protein we noted greater (magnitude) and more prolonged (duration) overall tyrosyl phosphorylation relative to cells expressing SHP-1^mAID^ alone (**Figures 2C, E**, compare left and right panels; representative examples of increased and prolonged phosphorylation are indicated by red arrows I–III). Enhanced phosphorylation of LAT at pY191 was further confirmed using a phospho-specific antibody (**Figures 2C, E**). Cell viability and proliferation were unaffected by the mAID tag, with no differences observed between wild-type SHP-1 and SHP-1^mAID^ expressing cells (data not shown). Taken together, these data demonstrate that the C-terminal fusion of mAID does not impair SHP-1 function, and that auxin-induced, OsTIR1-dependent degradation of SHP-1^mAID^ leads to enhanced TCR signaling comparable to what has previously been reported for SHP-1-deficient T cell lines (22), validating the system for inducible and specific protein depletion in both murine and human T cells.

### Engineering a tissue-specific inducible and reversible *in vivo* protein degradation mouse model

To translate the *in vitro* validated IRIP system into a murine model, we generated mice in which the mAID tag was inserted at the C-terminus of the endogenous SHP-1 gene, creating the *SHP-1^mAID^* knock-in allele (**Supplementary Figure 1**). Successful expression of the SHP-1^mAID^ fusion protein was confirmed by immunoblotting with an anti-SHP-1 antibody in both lymph nodes and spleens, and SHP-1^mAID^ was expressed at levels comparable to wild-type SHP-1, indicating that the knock-in does not disrupt SHP-1 expression or stability under basal conditions (**Figure 3A**). These mice were crossed with a Cre-inducible OsTIR1 transgenic line in which OsTIR1 is knocked into the ROSA26 locus and expressed following Cre-mediated excision of a STOP cassette (**Figure 3B**). To restrict the system to the T cell lineage, Cre recombinase expression was placed under the control of the T cell-specific distal Lck promoter (32, 42). Immunoblot analysis of T cells isolated from mice homozygous for *SHP-1^mAID^*, with or without *OsTIR1* and *Cre*, confirmed that SHP-1^mAID^ protein levels were equivalent across all conditions in the absence of auxin, establishing that OsTIR1 and Cre expression alone are not sufficient to drive SHP-1^mAID^ degradation and validating the specificity for conditional *in vivo* degradation (**Figure 3C**).

**Figure 3:**
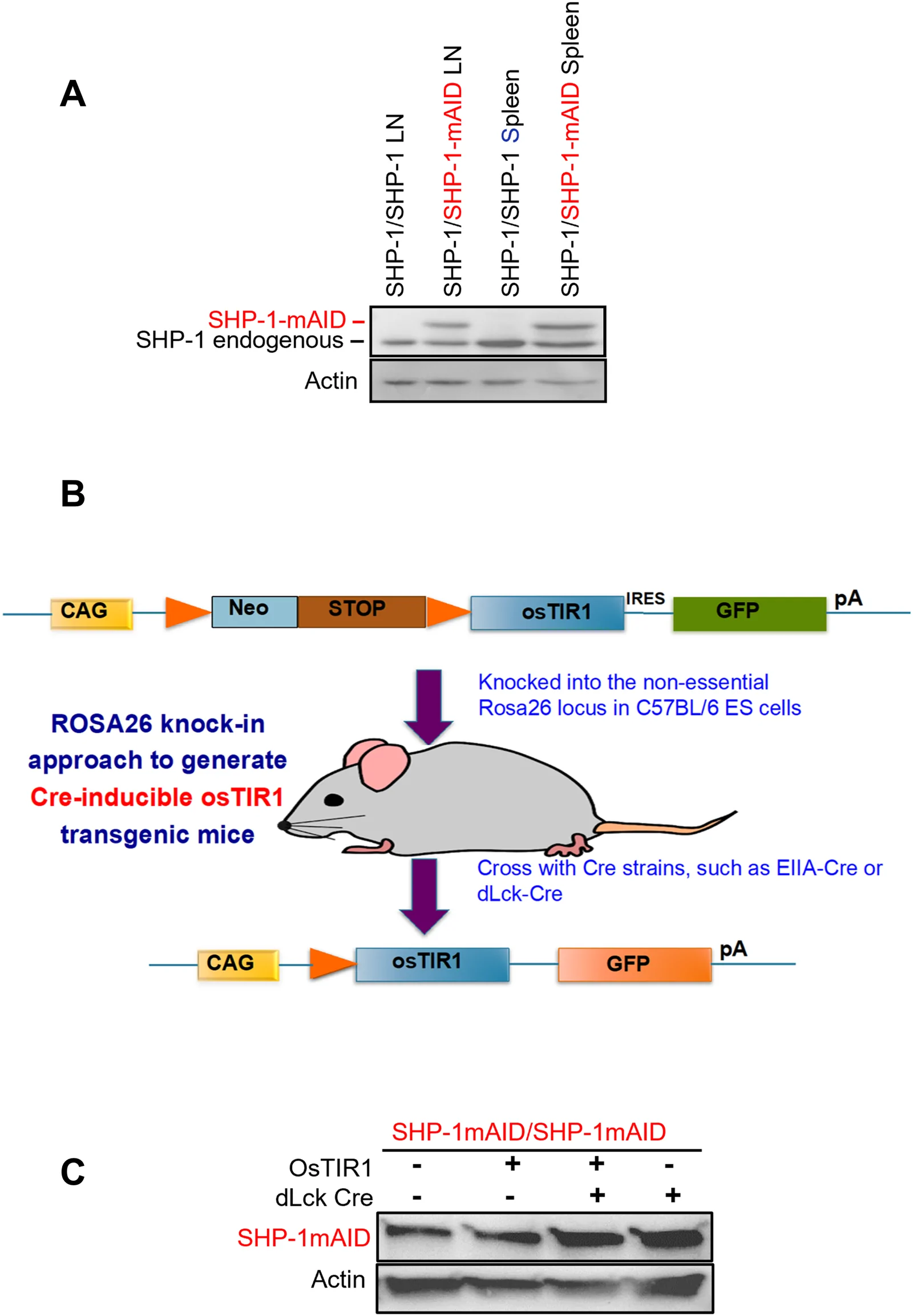
Generation of a murine model for inducible and reversible *in vivo* protein degradation (IRIP). **(A)** Immunoblot analysis of SHP-1 protein levels in spleen and lymph node (LN) cells from mice expressing wild-type SHP-1 or SHP-1^mAID^, showing comparable expression of endogenous and mAID-tagged protein. **(B)** Schematic of the IRIP mouse model. A knock-in construct with a Cre-inducible *OsTIR1* transgene was targeted to the *ROSA26* locus in C57BL/6 embryonic stem (ES) cells. These knock-in mice were further crossed with Cre-expressing strains (e.g., *EIIa-Cre* for ubiquitous expression, or *dLck-Cre* for T-cell specific expression) **(C)** SHP-1 expression in T cells isolated from mice homozygous for the *SHP-1mAID* allele, with or without co-expression of *OsTIR1*, demonstrating basal expression is unaffected. Data are representative of three independent experiments.

### Kinetics, specificity and reversibility of the SHP-1^mAID^ degradation system *in vivo*

We next evaluated the dose response, kinetics, and specificity of SHP-1^mAID^ degradation *in vivo*. Unless otherwise indicated, the mice used had three different engineered loci: *SHP-1*^mAID^ (homozygous):*OsTIR1+:dLck-Cre+*. Because the auxin used in the *in vitro* studies (obtained from RPI) was soluble only in DMSO, which is not suitable for *in vivo* administration, we switched to a PBS-soluble auxin formulation (Cayman Chemical). As this formulation showed substantially lower biological activity (**Supplementary Figure 3**), higher concentrations were required for *in vivo* use. To determine the effective dose, mice received once-daily injections of auxin at 200, 400, or 600 mg/kg for three consecutive days. Splenic T cells showed dose-dependent SHP-1^mAID^ degradation, with complete loss observed at 600 mg/kg, confirming that the system is tunable *in vivo* (**Figure 4A**; **Supplementary Figure 4A**). To characterize degradation kinetics, mice received daily injections at 600 mg/kg for 1 to 3 consecutive days. SHP-1^mAID^ levels declined progressively from day 2 onward, with complete degradation achieved after three injections (**Figure 4B**; **Supplementary Figure 4B**). To confirm that degradation required both OsTIR1 and Cre, auxin was administered to mice expressing SHP-1^mAID^ but lacking either Cre or OsTIR1. No SHP-1 degradation was observed in these animals, whereas efficient degradation occurred in *SHP-1^mAID:^ OsTIR1+:dLck-Cre+* T cells, establishing strict dependence on Cre-driven OsTIR1 expression (**Figure 4C**; **Supplementary Figure 4C**). The selectivity of auxin-induced degradation for the tagged allele was further confirmed in mice heterozygous for *SHP-1^mAID^*, in which auxin induced loss of the mAID-tagged SHP-1 while the untagged endogenous protein remained fully stable (**Figure 4D**; **Supplementary Figure 4D**).

**Figure 4:**
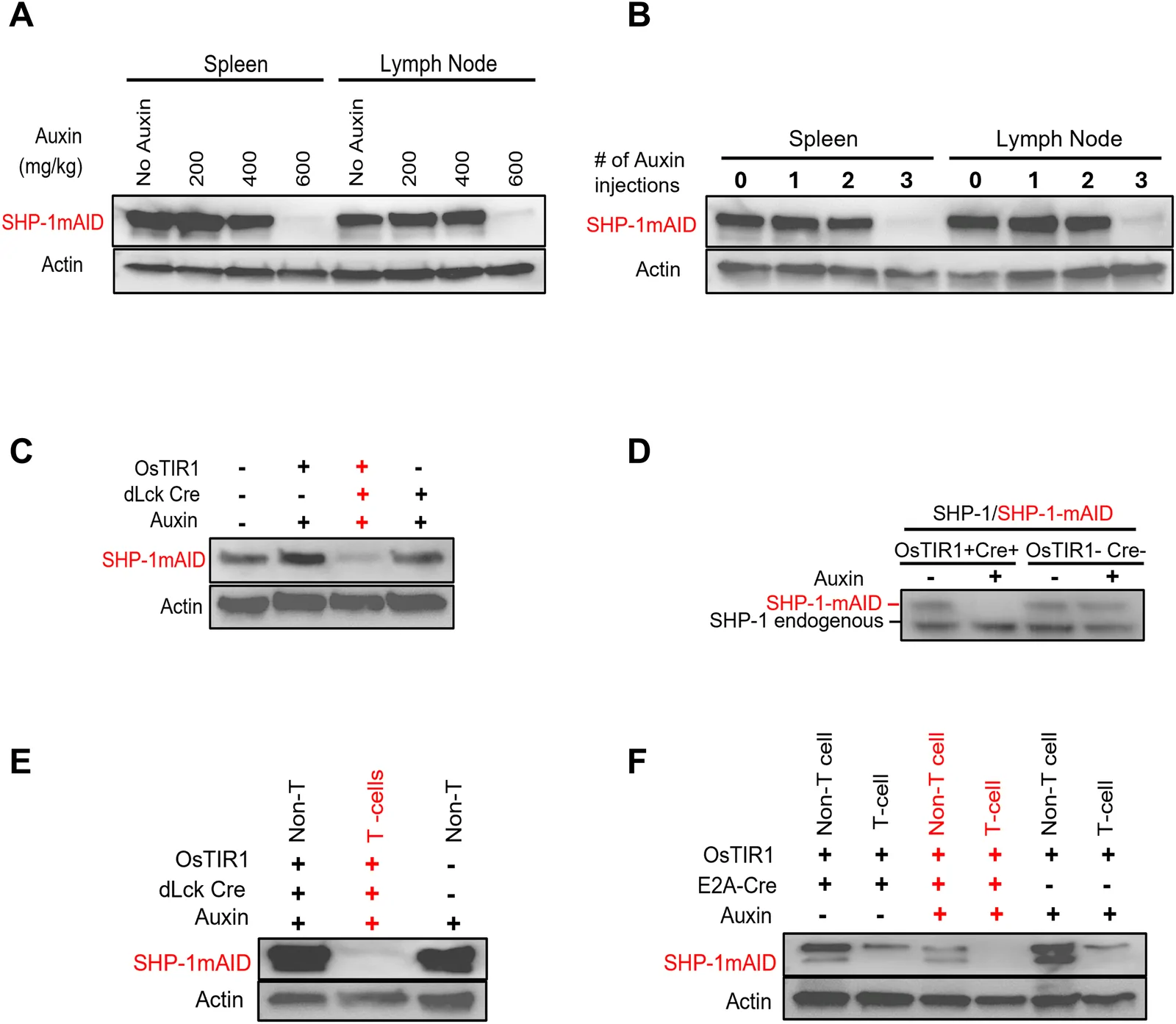
Validation of the IRIP system: Functional kinetics and specificity of SHP-1mAID depletion **(A)** Dose-dependent degradation of SHP-1mAID in T cells from mice injected once/day for three consecutive days with the indicated auxin doses. **(B)** Kinetics of SHP-1mAID degradation in T cells from *SHP-1mAID*:*OsTIR1^+^:dLck-Cre^+^* mice after 1–3 daily injections of auxin at 600 mg/kg. Cell lysates were analyzed by immunoblotting for SHP-1 and Actin. **(C)** SHP-1 degradation is OsTIR1-dependent. Cell lysates from mice of different genotypes and following selected dose and duration of auxin administration were analyzed by immunoblotting using antibodies against SHP-1 and actin. **(D)** SHP-1 degradation is mAID dependent. Cell lysates from mice heterozygous for *SHP-1mAID*, injected with selected auxin doses and durations were analyzed by immunoblotting using antibodies against SHP-1 and actin. **(E)** Tissue specificity of degradation. Cell lysates from T-cells and non-T-cells from *SHP-1mAID:OsTIR1+:dLck-Cre+* mice following auxin treatment, were analyzed by immunoblotting using antibodies against SHP-1 and actin. **(F)** The IRIP system is functional in other cell lineages. Cell lysates from T- cells and non-T-cells derived from *SHP-1mAID:OsTIR1+:EIIA-Cre+* and various control mice with and without auxin treatment, were analyzed by immunoblotting using antibodies against SHP-1 and actin. All immunoblots are representative of three independent experiments. β-actin protein levels serve as loading controls.

To assess cell-lineage specificity, T cells and non-T cells were isolated from the spleens of *SHP-1^mAID:^ OsTIR1+: dLck-Cre+* mice following auxin treatment. Degradation occurred exclusively in T cells, with no detectable loss of SHP-1^mAID^ in non-T cells (**Figure 4E**; **Supplementary Figure 4E**), consistent with *dLck*-Cre-mediated OsTIR1 expression being restricted to the T cell lineage. To explore whether the system could be extended to other lineages, *SHP-1^mAID:^ OsTIR1+* mice were crossed with the ubiquitously expressing *EIIA*-Cre deleter strain (33). Despite the substantially higher basal expression of SHP-1 in non-T cells, auxin-induced SHP-1^mAID^ degradation was observed in both T and non-T cell populations in these mice (**Figure 4F**; **Supplementary Figure 4F**), demonstrating that broader OsTIR1 expression expands the range of cell types amenable to IRIP-mediated depletion. Together, these results establish that *in vivo* SHP-1^mAID^ degradation is tunable, strictly OsTIR1-dependent, and can be directed to selected cell lineages through the choice of Cre driver.

### Temporary *in vivo* depletion of SHP-1 drives reversible T cell hyperproliferation

While SHP-1 is widely recognized as a negative regulator of TCR-mediated signaling, specifically ascribing its role has remained controversial, i.e. whether the hyperproliferation observed in primary T cells from SHP-1-deficient mice reflects a cell-intrinsic effect of SHP-1 loss at the time of TCR stimulation (22, 25), or an imprinted consequence of SHP-1 deficiency during an earlier stage of T cell development (30). The IRIP system is uniquely suited to address this question, as it allows SHP-1 deficiency to be induced acutely and transiently within a defined temporal window. Using the conditions established above for complete SHP-1^mAID^ degradation, we first characterized the *in vivo* kinetics of protein re-expression following auxin administration and found partial re-expression at 36 hours and full re-expression by 60 hours after the last injection (**Figure 5A**; **Supplementary Figure 5**).

**Figure 5:**
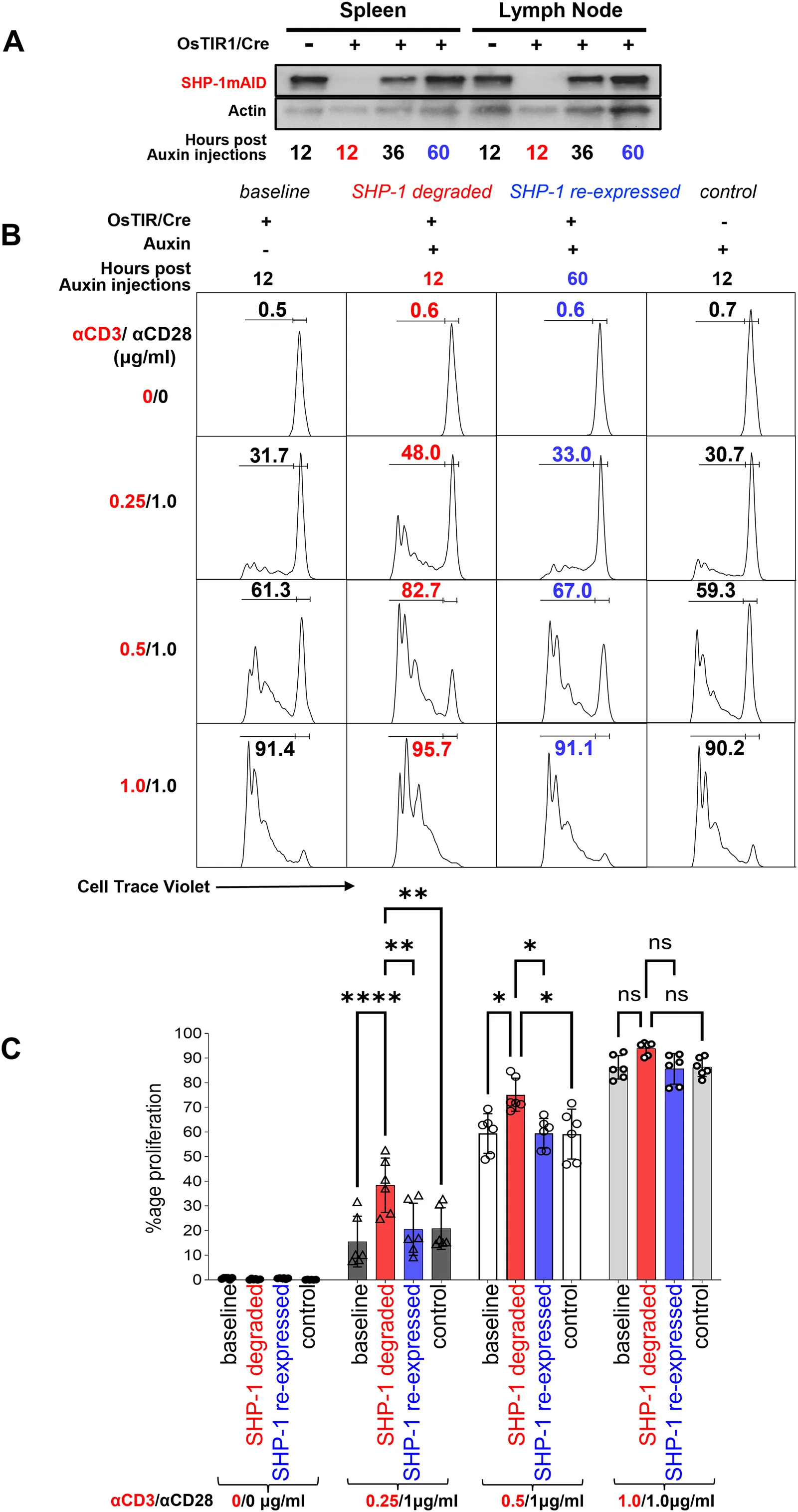
Functional assessment of cell-intrinsic T cell responses following reversible, auxin-induced SHP-1^mAID^ degradation. **(A)** *SHP-1^mAID^:OsTIR1+:dLck-Cre+* and control mice were for three consecutive days once/day injected with auxin (600 mg/kg) or PBS. At the indicated times after the last injection, splenic and lymph node derived T cells were isolated, lysed and analyzed for SHP-1 levels by Western Blot, which shows the kinetics of SHP-1 protein re-expression. **(B, C)** auxin-induced SHP-1 degradation leads to T cell hyper-proliferation. CD4+ T cells isolated from the indicated mice were Cell Trace Violet (CTV) labeled, stimulated *in vitro* with indicated concentrations of plate-bound anti-CD3 and anti-CD28 for 72 hours. Proliferation was analyzed by CTV dilution by flow cytometry. **(B)** Representative histograms show hyper-proliferation in cells from auxin-treated *SHP-1^mAID^:OsTIR1+:dLck-Cre+* mice, which reverses as SHP-1 is re-expressed. The numbers indicate the percentage of divided cells. Data are representative of 3 independent experiments. **(C)** Graphical representation showing the statistical analysis of the proliferation assay.

We then assessed *ex vivo* proliferation of splenic T cells isolated from *SHP-1^mAID:^ OsTIR1+:dLck-Cre+* mice either 12 hours after auxin injection when SHP-1 was fully degraded, or 60 hours after auxin injection when SHP-1 was fully re-expressed. Control groups consisted of *SHP-1*^mAID:^ *OsTIR1+:dLck-Cre+* mice injected with PBS and *SHP-1*^mAID^ mice lacking *OsTIR1* and *dLck-Cre* injected with auxin, both harvested at 12 hours. T cells from SHP-1-depleted mice showed robust hyperproliferation upon anti-CD3/anti-CD28 stimulation, most pronounced under suboptimal anti-CD3 conditions (**Figures 5B, C**), consistent with previous findings from our group (22, 29) and others (16, 43, 44). Critically, this hyperproliferative response was fully reversed to levels comparable to controls in mice from which SHP-1 had been allowed to re-express (**Figures 5B, C**). These findings directly demonstrate that SHP-1 restrains T cell proliferation in a cell-intrinsic manner downstream of TCR engagement, independently of any developmental or environmental preconditioning. Beyond resolving this long-standing question about SHP-1 function, these results illustrate the broader utility of the IRIP system as a platform for dissecting the acute physiological roles of proteins in a tissue-specific manner, free from the confounding effects of developmental adaptation.

## Discussion

To study the acute, cell-intrinsic functions of signaling proteins in T cells, we developed the Inducible and Reversible *in vivo* Protein Degradation (IRIP) system, a platform that combines auxin-induced degron technology with Cre-driver-mediated, lineage-specific expression of OsTIR1, enabling rapid, highly specific, and reversible depletion of endogenously tagged proteins in defined cell populations. By applying this system to SHP-1, a well-established negative regulator of T cell receptor signaling, we demonstrate that SHP-1 directly and cell-intrinsically restrains T cell proliferation in an acutely operative manner, resolving a long-standing controversy in the field.

Several features distinguish IRIP from currently available tools for studying protein function in T cells. Unlike constitutive gene deletion, which exposes cells to permanent protein loss throughout development and allows for compensatory rewiring of signaling networks, IRIP restricts protein depletion to a defined and controlled temporal window. This is a critical advantage, as chronic absence of a signaling protein can fundamentally alter the transcriptional, epigenetic, and signaling landscape of a cell in ways that are entirely unrelated to the acute function of that protein (45, 46). Unlike RNA interference approaches, IRIP operates at the level of the endogenous protein and does not rely on transfection or transduction, avoiding the associated efficiency and off-target concerns. Unlike pharmacological inhibitors, which often lack sufficient selectivity and can affect related family members or unintended targets, auxin-induced degradation is strictly dependent on the co-expression of OsTIR1 and the presence of the mAID tag, neither of which has any endogenous counterpart in mammalian cells. Moreover tissue-specific expression of OsTIR1 supports additional selectivity. The growing family of targeted protein degradation technologies, including proteolysis-targeting chimeras (PROTACs) and other proximity-induced degradation platforms, has demonstrated the broad utility of controlling protein levels post-translationally rather than at the transcriptional or translational level (47–50). IRIP extends this paradigm into the *in vivo* immunological setting with a level of lineage specificity and reversibility that many of these other approaches do not currently offer. While the first generation AID system we used in this study has proven informative in the context of SHP-1 and the scientific questions posed, newer, recently developed second generation auxin-inducible degron systems, such as the super-sensitive AID (ssAID/AID2), which incorporates an F74A or F74G point mutation in OsTIR1, demonstrate dramatically enhanced ligand sensitivity (51, 52). Although we detected no overt auxin-induced toxicity or basal ligand-independent degradation with our current setup, implementing these upgraded AID platforms can further minimize potential off-target and basal degradation effects.

A key design consideration was ensuring that the mAID tag itself did not perturb the biology under study. We addressed this comprehensively by demonstrating that C-terminal fusion of mAID to SHP-1 did not alter its expression level, stability, or ability to regulate TCR-proximal tyrosine phosphorylation in either murine BYDP or human Jurkat T cells. The pattern and kinetics of TCR/CD4-induced tyrosine phosphorylation were indistinguishable between wild-type and SHP-1^mAID^ expressing cells in the absence of auxin, and cell viability and proliferation were unaffected. This is consistent with prior work showing that the C-terminus of SHP-1 tolerates domain additions without loss of catalytic or regulatory function (38, 53), and provides confidence that phenotypes observed upon auxin-induced degradation reflect the loss of SHP-1 activity rather than any artifact introduced by the tag.

The *in vivo* implementation of IRIP required several adaptations relative to the *in vitro* system. The PBS-soluble auxin formulation used for animal studies showed substantially lower biological activity than the DMSO-soluble auxin used in cell culture, necessitating higher doses and multiple consecutive injections to achieve complete protein depletion. No overt toxicity was observed at the doses used, and the *in vivo* system performed robustly: degradation was dose- and injection-number-dependent and reversible with fully restored protein expression within 2–3 days following the last auxin injection. Moreover, the auxin-inducible degradation strictly required co-expression of OsTIR1 and Cre and was confined to the T cell lineage under dLck-Cre while remaining extendable to other lineages through alternative Cre drivers. The latter point is particularly noteworthy, as it demonstrates that IRIP is not intrinsically T cell-restricted, rather a generalizable platform whose lineage specificity is entirely determined by the choice of Cre driver, making it applicable to any cell type for which a validated Cre strain exists.

The functional experiments using IRIP directly addressed a controversial question in the field of SHP-1 biology. T cell-specific constitutive SHP-1 knockout mice, including those generated using dLck-Cre, display T cell hyperproliferation particularly under suboptimal TCR stimulation conditions, a phenotype that has been reported by multiple groups including our own (22, 25). However, because SHP-1 is absent throughout T cell development in these models, including during thymic selection, peripheral homeostasis, and prior antigen experience, it has been impossible to determine whether the hyperproliferative phenotype observed upon *in vitro* restimulation is a direct consequence of SHP-1 loss at the moment of TCR engagement, or whether it is instead an imprinted property of T cells that developed and were conditioned in the chronic absence of SHP-1 (30). These two possibilities have meaningfully different implications: the former would place SHP-1 as a dynamically operative and acutely relevant brake on TCR signal amplitude, while the latter would suggest that the observed phenotype is largely a reflection of altered developmental programming rather than acute signaling regulation. Our results unambiguously favor the former interpretation. T cells in which SHP-1 was acutely depleted in adult mice showed robust hyperproliferation upon restimulation, recapitulating the phenotype of constitutive knockouts. Critically, when SHP-1 was allowed to re-express over the subsequent 60 hours, proliferative responses returned fully to baseline levels. This reversibility is the key piece of evidence: it demonstrates that the hyperproliferative state is not a stable imprinted property of the T cells but rather a direct and dynamic consequence of the presence or absence of SHP-1 protein at the time of TCR stimulation. These findings are fully consistent with the established biochemical role of SHP-1 in dephosphorylating TCR-proximal substrates including ZAP-70, LAT, and Lck, thereby attenuating signal propagation downstream of the TCR (54–56), and place SHP-1 as an actively operative and physiologically relevant negative regulator of T cell activation rather than primarily a developmental checkpoint.

The broader implications of this work extend beyond SHP-1 biology. Many signaling proteins in immunology are studied exclusively through constitutive or inducible knockout models, and the interpretation of resulting phenotypes is routinely complicated by the possibility of developmental adaptation, compensatory upregulation of related family members, or secondary effects arising from altered cell composition or homeostasis. The IRIP system offers a means to bypass these complications by restricting protein loss to a defined window in adult, fully differentiated cells, and by allowing the phenotype to be reversed as an internal control for specificity. This reversibility feature is particularly powerful, as it provides within-experiment evidence that observed phenotypes are directly caused by the loss of the target protein rather than by unintended consequences. Future applications of IRIP could include dissecting the acute roles of other phosphatases, kinases, or adaptor proteins in T cell activation, anergy, exhaustion, or memory formation, as well as extending the platform to other immune cell lineages such as B cells, macrophages, or dendritic cells through the appropriate choice of Cre driver. Furthermore, recent studies have demonstrated that the scope of the AID system extends beyond cytosolic targets to include nuclear and membrane-bound proteins, including receptors, provided the degron tag remains accessible to the intracellular E3 ubiquitin ligase complex (51). When tagging the intracellular domain of a membrane protein is unfeasible, alternative degradation platforms can target extracellular domains, though these systems generally lack tissue specificity (57). The IRIP system could also in principle be applied to study protein function during specific phases *in vivo* immune responses, including infection, autoimmunity, or tumor immunity, by timing auxin administration to coincide with defined stages of the response.

In summary, we have developed and validated IRIP as a robust, specific, reversible, and lineage-flexible platform for acute protein depletion *in vivo*. Applied to SHP-1, IRIP provides the clearest evidence to date that SHP-1 functions as a direct, cell-intrinsic, and acutely operative negative regulator of T cell proliferative responses downstream of the TCR. These findings resolve a long-standing controversy in T cell biology and establish IRIP as a broadly useful tool for the immunology community.

## Data Availability

The original contributions presented in the study are included in the article/**Supplementary Material**, further inquiries can be directed to the corresponding author/s.
