# Supplementary material for: Inducible and reversible in vivo protein degradation reveals T cell-intrinsic regulatory function of SHP-1
Source: Front Immunol. 2026 Oct 21;17:1961691. doi: 10.3389/fimmu.2026.1961691 (PMC13731291; doi:10.3389/fimmu.2026.1961691)
Supplement: Supplementary file 1 [file Supplementaryfile1.pdf]

A

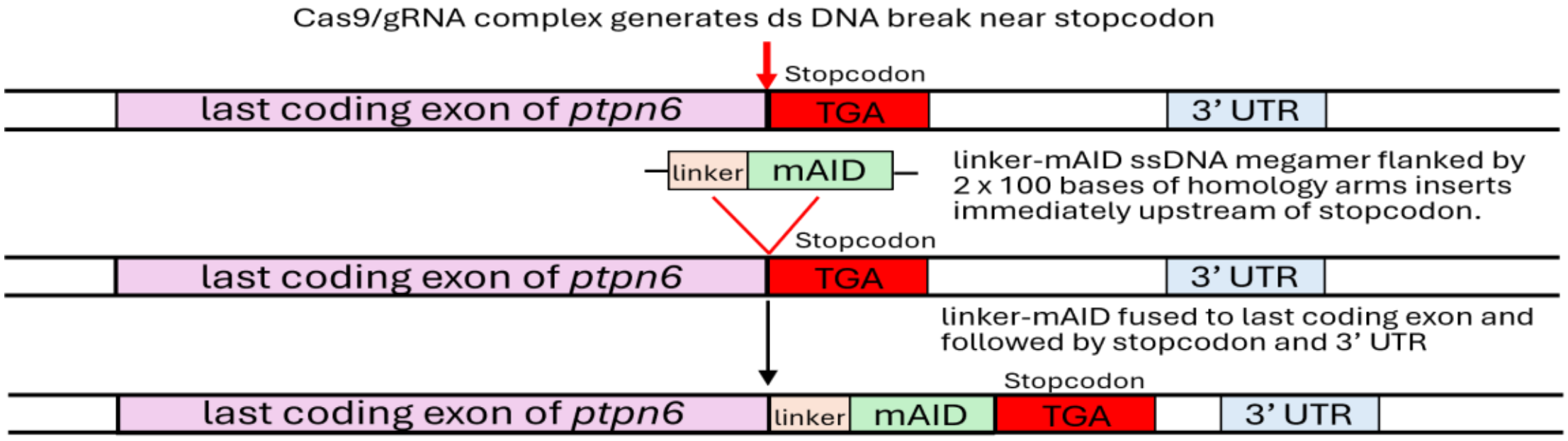

B

mAID

mAID amino acid Sequence:
KEKSACPKDPAKPPAKAQVVGWPPVRSYRKNVMVSCQKSSGGPEAAAFVKVSMDGAPYLRKIDLRMYK*

Linker amino acid sequence:
GAGAGAGAGA
gRNA nucleotide sequence for B6 mice: TCTCTCAAGAGGAAGTGATC TGG   Red indicate PAM sequence

Combined nucleotide sequence:
GAGGTGTACGAAAACGTGCATAGCAAGAGCAAGAAGGAAGAGAAAGTAAAGAAGCAGCGGTCGGCAGACAAGGA
GAAGAACAAAGGTTCTCTCAAGAGGAAGGGTGCAGGCGCCGGGGCTGGTGCTGGGGCAAAGGAGAAGAGTGCT
TGTCCTAAAGATCCAGCCAAACCTCCGGCCAAGGCACAAGTTGTGGGATGGCCACCGGTGAGATCATACCGGAA
GAACGTGATGGTTTCCTGCCAAAAATCAAGCGGTGGCCCGGAGGCGGCGGCGTTCGTGAAGGTATCAATGGACG
GAGCACCGTACTTGAGGAAAATCGATTTGAGGATGTATAAAtgaTCTGGGCATTCGTCTGCAGGTGGCCATGGTGCG
GCCCCTTCTGTGTGTTCTTTCTCTCCCTGCCCCCATTTCCCCAACTTCAGTTCAGACGTGCGTGCATGG

BLACK: flanking sequence
RED: linker
BLUE: mAID sequence

C

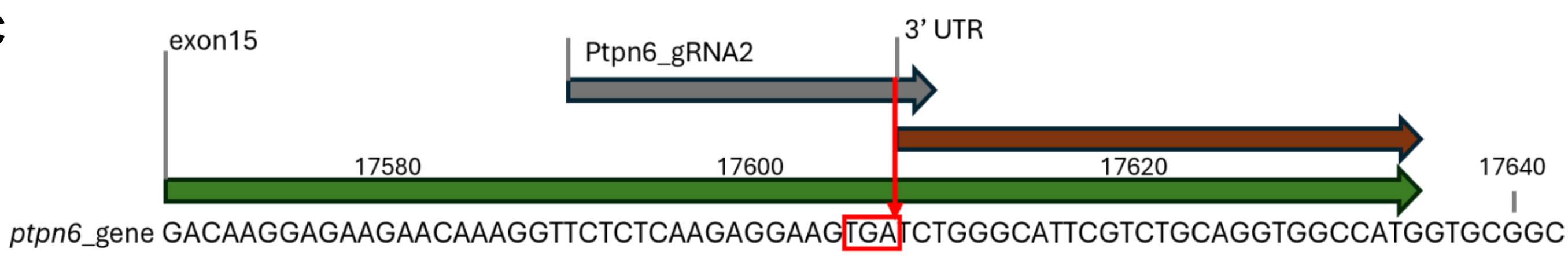

D

PCR primers for genotyping

SHP-1
Ptpn6_FW1: GGAGTCGGAGTAATGGAATA
Ptpn6_FW1: TTACAAGAAGATGGGGGTGG
Product size: WT 836bp, KI 1076bp

OsTIR1 (ROSA26-YFP vector)
ROSA-YFP1 (Jax YFP 8545) - 5- AAA GTC GCT CTG AGT TGT TAT -3
ROSA- YFP2 (Jax 4982) - 5- AAG ACC GCG AAG AGT TTG TC -3
ROSA YFP3 (Jax 8546) - 5- GGA GCG GGA GAA ATG GAT ATG – 3
Product size: WT 603bp, KI 842bp

oIMR8545 = common Rosa26 primer
oIMR8546 = WT Rosa26 primer
oIMR 4982 = YFP mutant primer

Size of the expected PCR products
Wild-type Rosa26 allele: ~600 bp
Rosa26-YFP allele (mutant/transgene): ~320 bp
Heterozygote: both bands present

Supplementary Figure 1:
(**A**) CRISPR-mediated C-terminal tagging of endogenous *ptpn6* with the minimal auxin-inducible degron (mAID) via HDR-directed ssDNA megamer insertion. (**B**) Amino acid and nucleotide sequences of the linker-mAID knock-in cassette with flanking homology sequence, linker, mAID coding sequence and the gRNA target site. (**C**) Genomic map of gRNA target site and the Cas9 cut position flanking the stop codon of the target locus for precision knock-in tag insertion. (**D**) PCR primers for indicated genotyping.

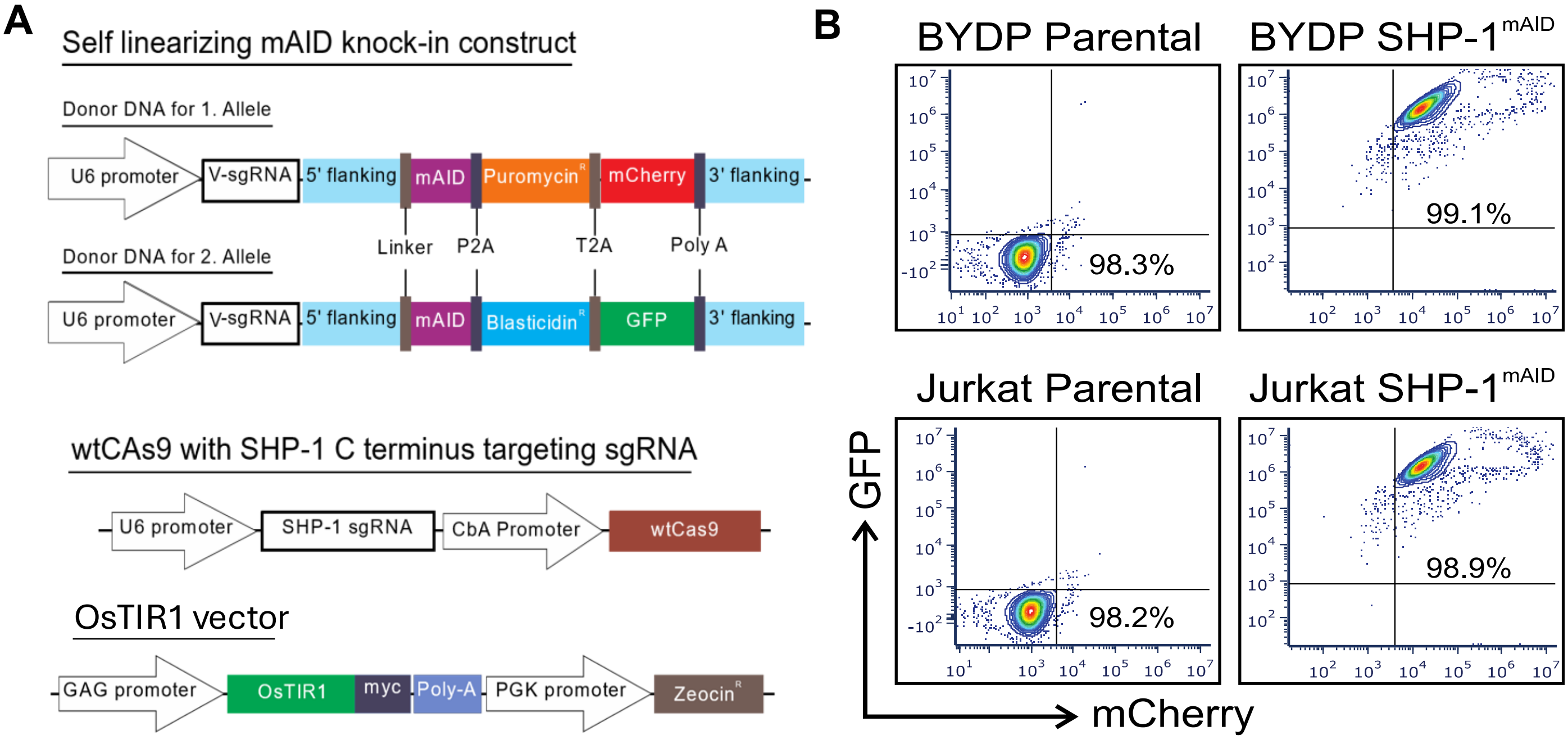

C

SHP-1 C terminus targeted sgRNA for BYDP (murine)

sgRNA sequence #1 GAGAGAAAGAACACACAGAA

sgRNA sequence # 2 GTCTGCAGGTGGCCATGGTG

SHP-1 C terminus targeted sgRNA for Jurkat (human)

sgRNA sequence #1 GTGCTGTCCTCAGGTGGCCA

sgRNA sequence #2 GCACCGCTCACTTCCTCTTG

Vector self cleavage sgRNA

sgRNA sequence GCGATAAGTCGTGTCTTACC

Supplementary Figure 2:

Generation and validation of an auxin-inducible degron system for SHP-1 in murine and human T cell lines. (**A**) (**Top**) Design of the self-linearizing knock-in construct for dual-allele targeting of the SHP-1 locus with mCherry and GFP fluorescent reporters. (**Bottom**) CRISPR-Cas9 vector strategy for C-terminal tagging of SHP-1 with mAID and for constitutive OsTIR1 expression. (**B**) Flow cytometry analysis confirming successful dual-allele SHP-1-mAID knock-in, as evidenced by co-expression of GFP and mCherry in BYDP and Jurkat cell lines. (**C**) sgRNA sequences for murine (BYDP) and human (Jurkat) T cell lines as well as self cleavage sgRNA for increased targeting efficiency.

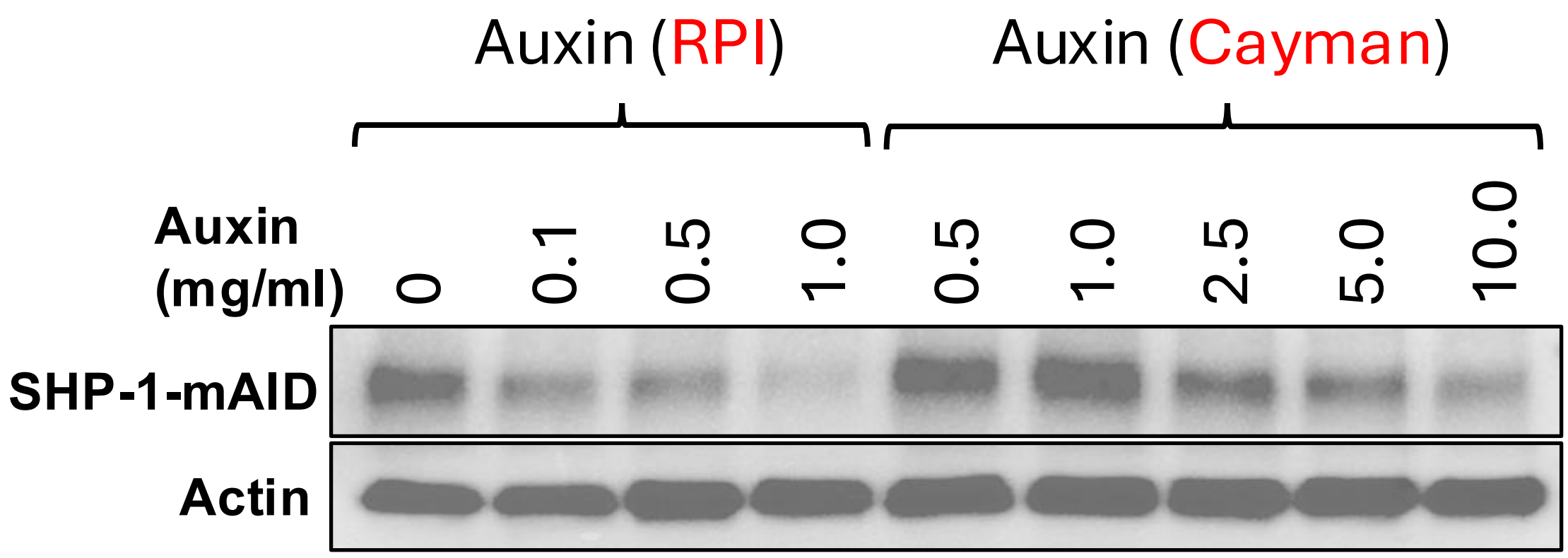

Supplementary Figure 3:

Comparison of the dose kinetics of the auxin-mediated SHP-1 protein degradation in murine splenocytes. T cells were isolated from the spleens of SHP^{mAID} E2A-Cre+Tg+ mice and treated *ex vivo* for 3 hours with the indicated doses of Auxin obtained from the respective sources. Equal aliquots of cellular lysates were analyzed by immunoblotting for SHP-1 and Actin protein expression.

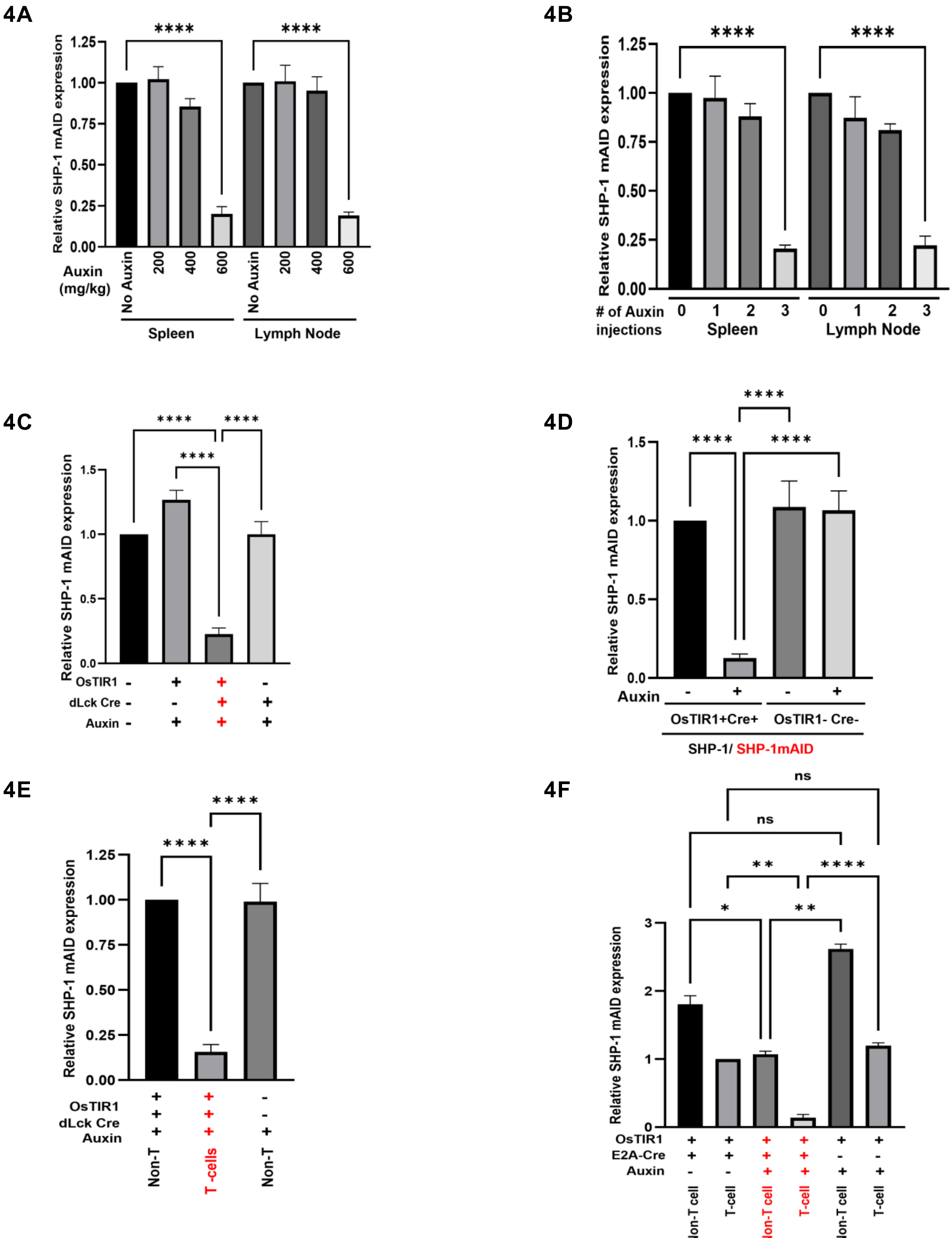

Supplemental Figure 4:

Quantitative densitometric analysis of complied Western Blot Images with corresponding representatives depicted in Figs. 4A-F. Band intensities were quantified using ImageJ software and normalized to β-actin. Data are representing three independent experiments. Statistical analysis was performed using one-way ANOVA to compare differences among the experimental groups. A p value of <0.05 was considered statistically significant, and all data are presented as mean ± SEM. Asterisks represent *p<0.05, **p<0.01, and ****p<0.0001.

5A

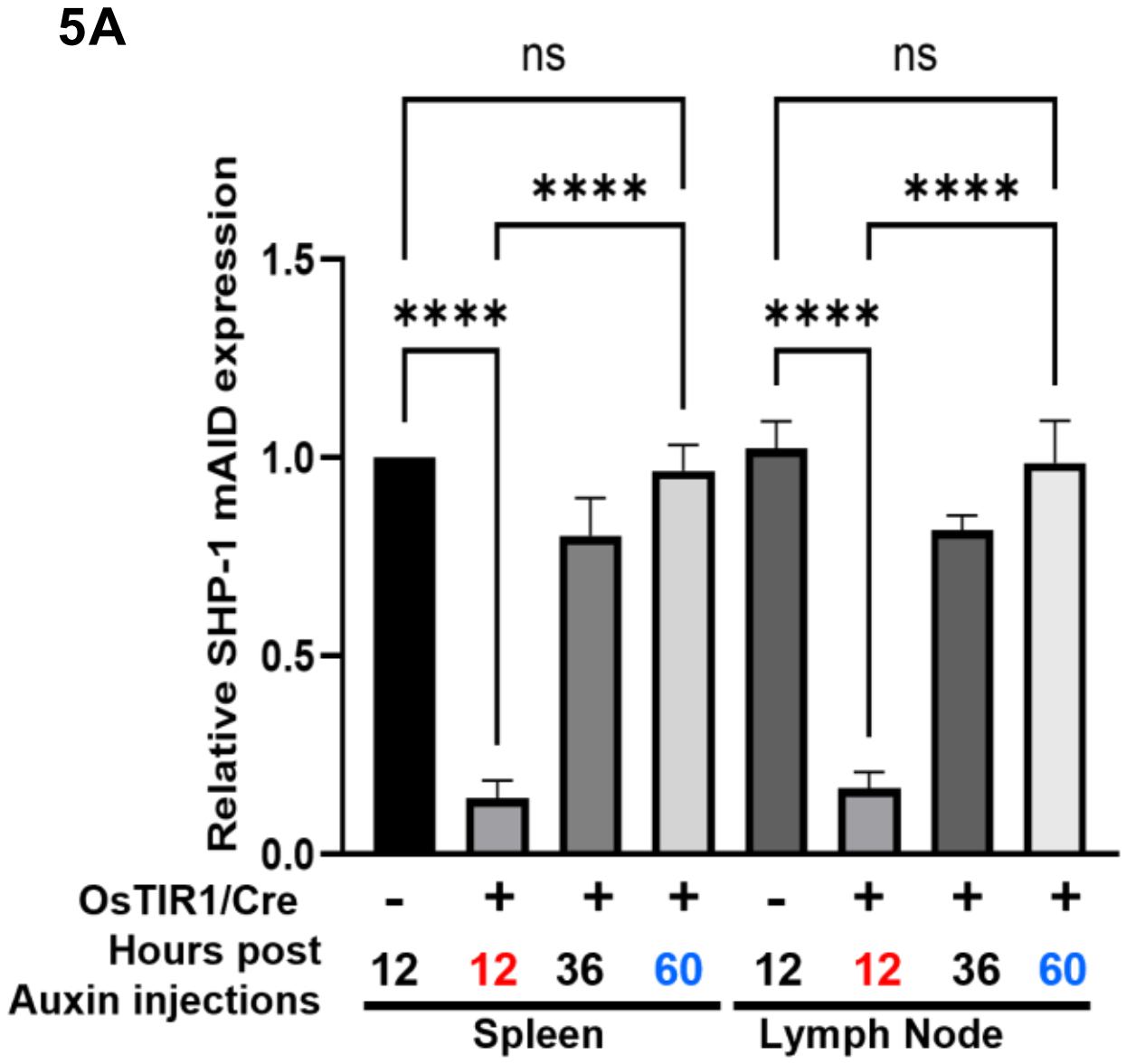

<u>Supplemental Figure 5:</u>

Quantitative densitometric analysis of complied Western Blot Images with corresponding representative depicted in Fig. 5A. Band intensities were quantified using ImageJ software and normalized to β-actin. Data are representing three independent experiments. Statistical analysis was performed using one-way ANOVA to compare differences among the experimental groups. A p value of <0.05 was considered statistically significant, and all data are presented as mean ± SEM. Asterisks represent ****$p<0.0001$.
